# *TYROBP* is a potential prognostic biomarker of clear cell renal cell carcinoma

**DOI:** 10.1002/2211-5463.12993

**Published:** 2020-10-31

**Authors:** Ping Wu, Tingting Xiang, Jing Wang, Run Lv, Guangzhen Wu

**Affiliations:** ^1^ Department of Anesthesiology The First Affiliated Hospital of Dalian Medical University China; ^2^ Department of Rehabilitation Liguang Rehabilitation Hospital of Dalian Development Zone China; ^3^ Department of Neurobiology Harbin Medical University China; ^4^ Anesthesiology Department Dalian Medical University China; ^5^ Department of Urology The First Affiliated Hospital of Dalian Medical University China

**Keywords:** bioinformatics analysis, biomarker, clear cell renal cell carcinoma, *HRG*, immunotherapy, *TYROBP*

## Abstract

Clear cell renal cell carcinoma (ccRCC) exhibits high recurrence and metastasis rates. Although target therapy has significantly improved the prognosis of some patients with ccRCC, the median survival rate remains poor. Thus, there remains a need for the identification of novel potential targets for diagnosis and therapy. Here, we screened differentially expressed genes between ccRCC and normal tissues through analyzing The Cancer Genome Atlas database. We identified 55 up‐regulated and 67 down‐regulated genes associated with poor prognosis. Gene Ontology and Kyoto Encyclopedia of Genes and Genomes pathway analysis revealed that these genes were associated with glycometabolic process, complement and coagulation cascades. In addition, the eight down‐regulated genes (*HRG*, *FABP1*, *ALDOB*, *PCK1*, *HAO2*, *CASR*, *PLG*, and *HMGCS2*) and two up‐regulated genes (*SERPINE1* and *TYROBP*) were filtered out. Finally, *TYROBP* was selected through repeated verification of various databases. High expression of *TYROBP* is associated with low survival rate in ccRCC, is closely related to immune cell infiltration and is coexpressed with Programmed cell death protein‐1(*PD‐1* *)* and Cytotoxic T lymphocyte‐associated antigen‐4(*CTLA‐4*). In conclusion, *TYROBP* may have potential for diagnosis and treatment of ccRCC.

## Graphical abstract

Clear cell renal cell carcinoma (ccRCC) exhibits high recurrence and metastasis rates. Although targeted therapy has significantly improved the prognosis of some patients with ccRCC, the median survival rate remains poor. In this article, we used a series of bioinformatics analyses to identify *TYROBP* as a potential target for diagnosis and treatment of ccRCC.

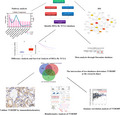

**Abbreviations**
BP: biological process
ccRCC: clear cell renal cell carcinoma
CTLA‐4: Cytotoxic T lymphocyte‐associated antigen‐4
DAVID: Database for Annotation, Visualization, and Integrated Discovery
DEG: differentially expressed gene
GO: Gene Ontology
IHC: immunohistochemistry
KEGG: Kyoto Encyclopedia of Genes and Genomes
KIRC: renal clear cell carcinoma
mDCs: mature dendritic cells
NK: natural killer
PAI‐1: Plasminogen activator inhibitor‐1
*PD‐1*: Programmed cell death protein‐1
PDL‐1: Programmed cell death 1 ligand 1
PPAR: peroxisome proliferator‐activated receptor alpha
PPI: protein–protein interaction
TCGA: The Cancer Genome Atlas

According to GLOBOCAN worldwide cancer statistics in 2018, it was estimated that approximately 400 000 new cases of kidney cancer were diagnosed, with a quarter of them succumbing to the disease [1]. Clear cell renal cell carcinoma (ccRCC) is the main pathological subtype (70–85%) of the primary renal tumor [2]. Because ccRCC lacks obvious early symptoms, nearly 30% of patients with ccRCC have already developed to the metastatic phase by the time of diagnosis [3]. Although early and localized ccRCC can be cured by partial or radical nephrectomy, nearly 30% of patients had postoperative recurrence and metastasis, called metastatic renal cell carcinoma [4]. In recent decades, molecular targeted therapy, represented by sorafenib, has been used in metastatic renal cell carcinoma with significant objective response rates, although the clinical outcomes are still unsatisfactory because of drug resistance and adverse effects [5]. Hence it becomes essential to explore underlying molecular mechanisms and identify novel targets for early diagnosis and precise treatment. Fortunately, as bioinformatics analysis has developed in recent years, we can deeply explore the potential genes and gene regulation networks through high‐throughput sequencing technology [6]. Currently, gene expression profiling has been applied to confirm genes involved in renal cell progression. By integrating different large databases to screen differentially expressed genes (DEGs) and constructing protein–protein interaction (PPI) networks, new biomarkers can be quickly identified and further validated. This makes it possible to investigate potential biomarkers for ccRCC and to explore related molecular mechanisms.

In this study, we first screened the DEGs from The Cancer Genome Atlas (TCGA) database. Subsequently, we conducted Gene Ontology (GO) and Kyoto Encyclopedia of Genes and Genomes (KEGG) pathway analysis to analyze DEGs enrichment pathways, which suggested that DEGs significantly engaged into glycometabolic process, complement and coagulation cascades, etc. Then PPI was used to identify intergenic interactions. Finally, we identified eight up‐regulated and two down‐regulated hub genes by using cytoHubba plug‐in in cytoscape. Furthermore, we performed differential expression analysis and survival analysis of hub genes through various databases. In addition, a meta‐analysis of data from six studies in the ONCOMINE database was used to verify the differential expression of hub genes. Through verification in multiple databases, the target gene *TYROBP* was finally identified. Because immunotherapy is currently a hot topic in renal cell carcinoma research, we conducted an immunological correlation analysis of *TYROBP* in the TIMER website(https://icbi.i‐med.ac.at/software/timiner/timiner.shtml), and we were surprised to discover that *TYROBP* has an intimated connection with immune cells infiltration and classic immune checkpoint gene (*PD‐1*, *CTLA‐4*). On the strength of this evidence, we speculated that *TYROBP* is likely to be an oncogene engaged in immune regulation and is a new key gene for ccRCC diagnosis and immunotherapy targeting.

## Materials and methods

### TCGA database

TCGA database was mined to screen for DEGs between ccRCC and kidney normal tissues from the UALCAN online tool (http://ualcan.path.uab.edu/index.html) [7]. The ONCOMINE database was used to further analyze and validate DEGs (https://www.oncomine.org/resource/main.html) [8]. The UALCAN database was used to screen the top 55 up‐regulated genes and top 67 down‐regulated genes with poor prognosis. We screened 80 up‐regulated genes and 80 down‐regulated genes from the ONCOMINE database and integrated the genes of the two databases through the VENE map.

### Gene Ontology and KEGG pathway analysis

The Database for Annotation, Visualization, and Integrated Discovery (DAVID; https://david.ncifcrf.gov/) is an online bioinformatics database for gene functional analysis [9], and the DAVID website plays a very important part in analyzing DEGs pathways. Therefore, we used DAVID to perform the GO function. In the KEGG pathway analysis, we used the CLUGO plug‐in in cytoscape to perform potential pathway analysis of DEGs (https://www.cytoscape.org/) [10, 11].

### PPI analysis and hub genes screening

The STRING website (https://string‐db.org/cgi/) was used to perform PPI analysis of DEGs [12], and we identified the hub genes by means of the CYTOHUBBA plug‐in in cytoscape [13, 14]. The top 10 hub genes with degrees >5 were selected.

### DEGs and survival outcome in ccRCC

We availed of the TCGA database (UALCAN website) and the ONCOMINE database to analyze the differential expression of hub genes in 533 ccRCC tissues and 72 normal kidney tissues. Then TCGA database (UALCAN website) and the Human Protein Atlas website (https://www.proteinatlas.org/) were used to analyze the relationship between these genes and survival rate [15].

### Immunohistochemistry

Samples of renal clear cell carcinoma and adjacent tissues of The First Affiliated Hospital of Dalian Medical University were selected for immunohistochemical analysis. All relevant patients signed informed consents. The study met with the approval of the Ethics Committee of The First Affiliated Hospital of Dalian Medical University, and the study methodologies conformed to the standards set by the Declaration of Helsinki. Paraffin pathological sections were first incubated for 2 h and then subjected to antigen retrieval. Pathological sections were stained with rabbit anti‐(human *TYROBP*) serum at 4 °C and then stained with horseradish peroxidase‐conjugated secondary antibody for 1 h. Immunohistochemistry (IHC) was performed using the 2,4‐diaminobutyric acid substrate kit, and finally, hematoxylin staining, sealing and photographing were performed. The analysis was performed using imagepro plus software, the semiquantitative analysis was performed using the integrated optical density/area method and statistics were performed using the graphpad prism 8.0 software (GraphPad Software, La Jolla California, USA). The data were expressed as mean ± standard deviation, and *P* < 0.05 was considered statistically significant.

### TIMER

TIMER is a comprehensive resource for systematic analysis of tumor‐infiltrating immune cells (https://icbi.i‐med.ac.at/software/timiner/timiner.shtml) [16, 17]. Therefore, we use the TIMER website to analyze the relationship between *TYROBP* and immune cell infiltration. Also, immunological correlation analysis was performed to observe the relevance between *TYROBP* and *PD‐1*, Programmed cell death 1 ligand 1(*PDL‐1*) and *CTLA‐4*.

### r Language analysis of *TYROBP* and *TYROBP*‐related genes

The RNA sequencing transcriptome data of the renal clear cell carcinoma(KIRC) cohort were downloaded from TCGA (https://cancergenome.nih.gov/) data portal. Then we used Limma package and pheatmap package to analyze the expression of *TYROBP* and genes that interact with *TYROBP* in 539 patients with tumor and 72 normal renal tissues. corrplot package of the r language (Lucent Technologies, USA) was availed of analyzing the coexpression of *TYROBP* and genes that interact with *TYROBP*.

## Results

### Identification of DEGs

**Fig. 1:**
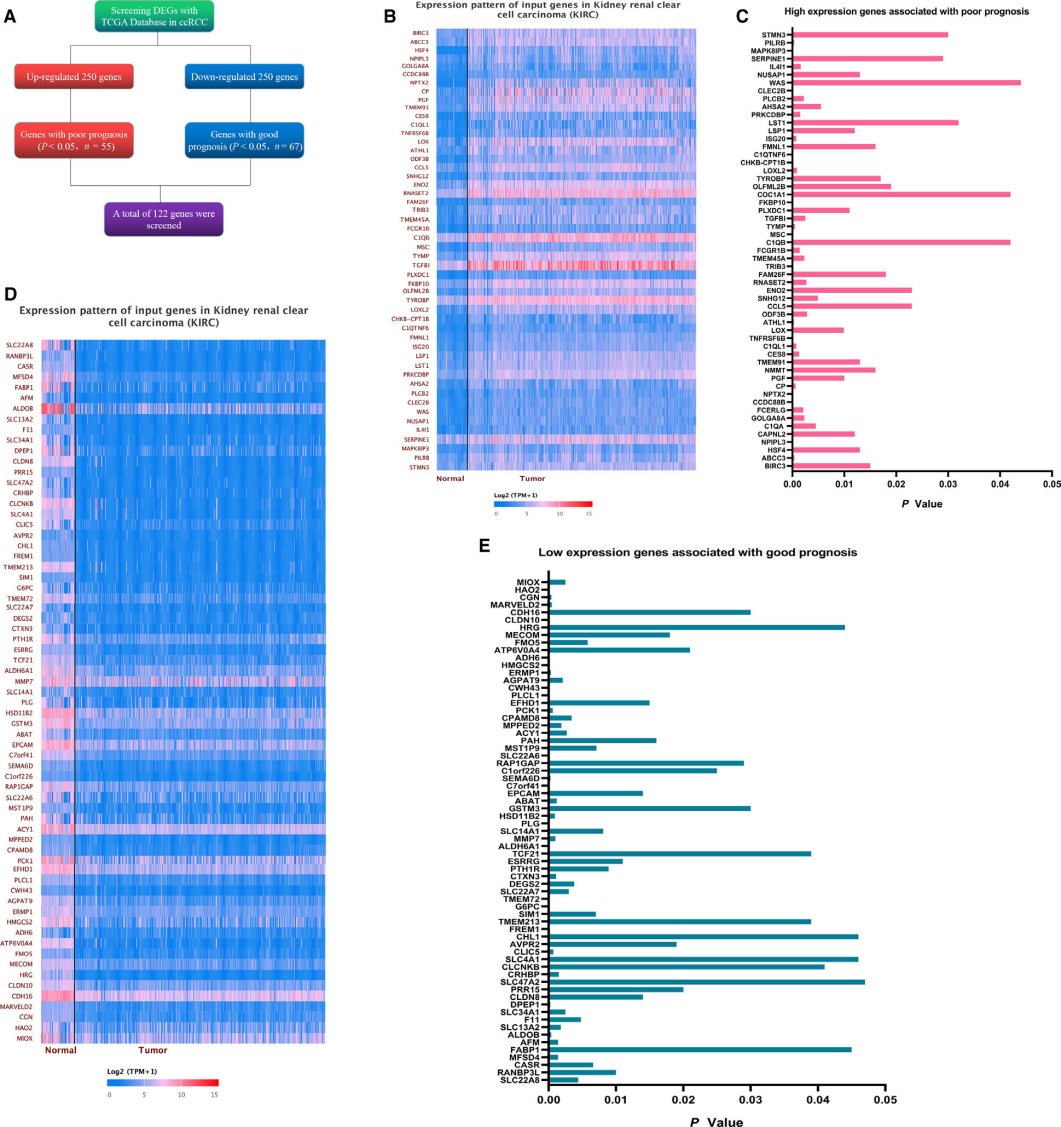
Screening for differential genes via TCGA database. (A) Screening for differential genes via the UALCAN online website. (B) The expression pattern of input genes with high expression in KIRC [the blue and red colors are log_2_(TPM + 1) (TPM, transcripts per million)]. The bluer the color of the heatmap, the lower the gene expression, and the redder the color of the heatmap, the higher the gene expression. (C) Screening of 55 genes with up‐regulated expression and related to poor prognosis in ccRCC compared with normal tissue (we used the paired *t*‐test for statistical analysis, and *P* < 0.05 was considered statistically significant). (D) The expression pattern of input genes with low expression in KIRC, and the *t*‐test was used to determine the *P* values [the blue and red colors are log_2_(TPM + 1)]. The bluer the color of the heatmap, the lower the gene expression, and the redder the color of the heatmap, the higher the gene expression). (E) Screening of 67 genes with down‐regulated expression and related to poor prognosis in ccRCC compared with normal tissue.

We used the UALCAN website to analyze the top 250 expressions of the up‐regulated and down‐regulated DEGs in TCGA database and then performed survival analysis on each gene separately. Finally, 55 genes with high expression and poor prognosis (Fig. 1B,C) and 67 genes with low expression and good prognosis were screened (Fig. 1D,E). A total of 122 DEGs were finally included (Fig. 1A and Table 1).

**Table 1:** A total of 122 DEGs were identified from TCGA datasets.

DEGs	Gene names
Up‐regulated (*n* = 55)	*BIRC3*, *ABCC3*, *HSF4*, *NPIPL3*, *CAPNL2*, *C1QA*, *GOLGA8A*, *FCERLG*, *CCDC88B*, *NPTX2*, *CP*, *PGF*, *NMMT*, *TMEM91*, *CES8*, *C1QL1*, *TNFRSF6B*, *LOX*, *ATHL1*, *ODF3B*, *CCL5*, *SNHG12*, *ENO2*, *RNASET2*, *FAM26F*, *TRIB3*, *TMEM45A*, *FCGR1B*, *C1QB*, *MSC*, *TYMP*, *TGFBI*, *PLXDC1*, *FKBP10*, *COC1A1*, *OLFML2B*, *TYROBP*, *LOXL2*, *CHKB‐CPT1B*, *C1QTNF6*, *FMNL1*, *ISG20*, *LSP1*, *LST1*, *PRKCDBP*, *AHSA2*, *PLCB2*, *CLEC2B*, *WAS*, *NUSAP1*, *IL4I1*, *SERPINE1*, *MAPK8IP3*, *PILRB*, *STMN3*
Down‐regulated (*n* = 67)	*SLC22A8*, *RANBP3L*, *CASR*, *MFSD4*, *FABP1*, *A FM*, *ALDOB*, *SLC13A2*, *F11*, *SLC34A1*, *DPEP1*, *CLDN8*, *PRR15*, *SLC47A2*, *CRHBP*, *CLCNKB*, *SLC4A1*, *CLIC5*, *AVPR2*, *CHL1*, *FREM1*, *TMEM213*, *SIM1*, *G6PC*, *TMEM72*, *SLC22A7*, *DEGS2*, *CTXN3*, *PTH1R*, *ESRRG*, *TCF21*, *ALDH6A1*, *MMP7*, *SLC14A1*, *PLG*, *HSD11B2*, *GSTM3*, *ABAT*, *EPCAM*, *C7orf41*, *SEMA6D*, *C1orf226*, *RAP1GAP*, *SLC22A6*, *MST1P9*, *PAH*, *ACY1*, *MPPED2*, *CPAMD8*, *PCK1*, *EFHD1*, *PLCL1*, *CWH43*, *AGPAT9*, *ERMP1*, *HMGCS2*, *ADH6*, *ATP6V0A4*, *FMO5*, *MECOM*, *HRG*, *CLDN10*, *CDH16*, *MARVELD2*, *CGN*, *HAO2*, *MIOX*

### GO and KEGG pathway analysis

**Fig. 2:**
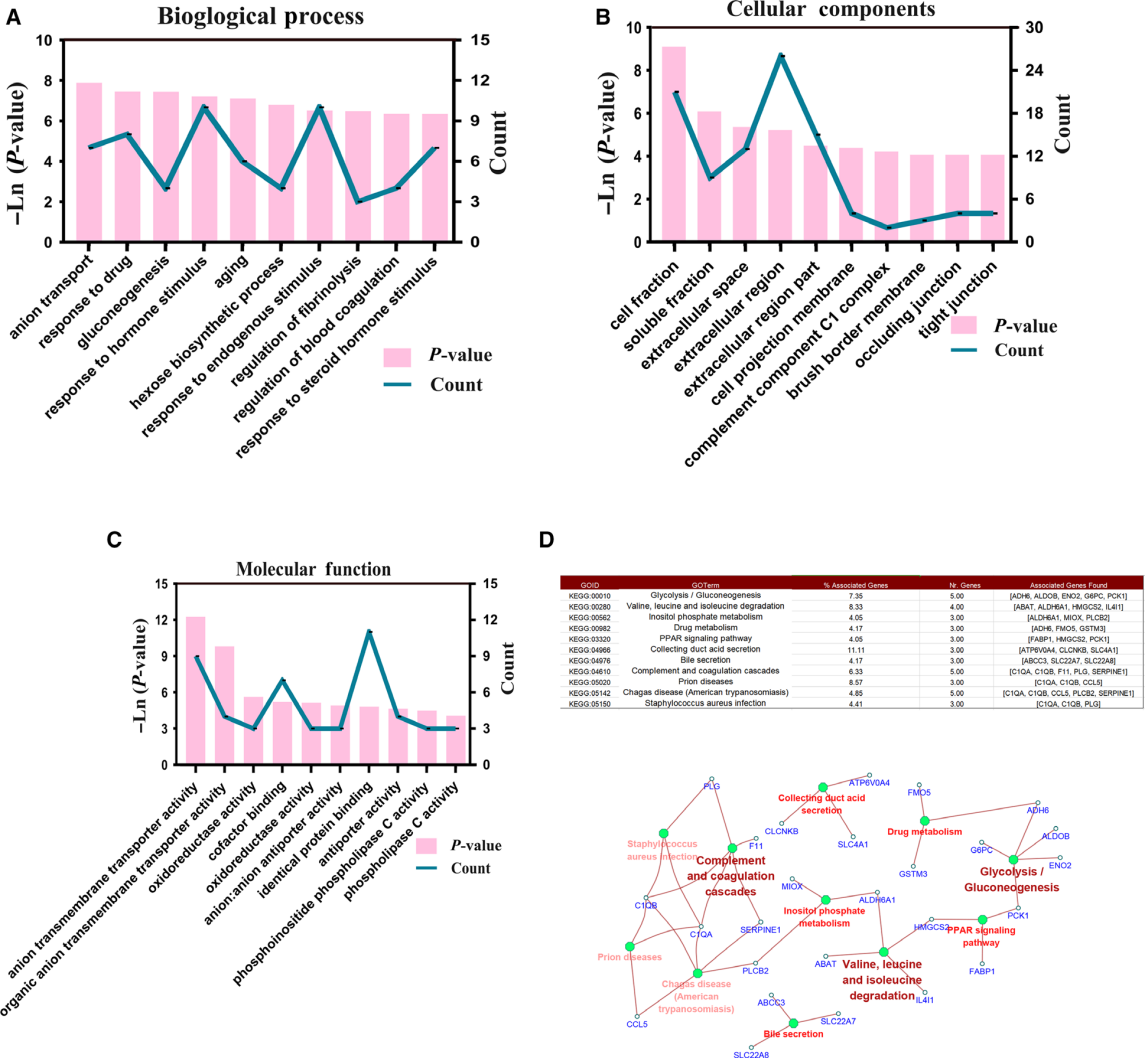
GO and KEGG pathway analysis through the DAVID website (we used the paired *t*‐test for statistical analysis, and *P* < 0.05 was considered statistically significant). (A) The top 10 enriched BPs of the DEGs based on GO analysis. (B) The top 10 enriched cellular component of the DEGs based on GO analysis. (C) The top 10 enriched molecular function of the DEGs based on GO analysis. (D) KEGG pathway analysis DEGs enrichment using CLUGO plug‐in in cytoscape software.

To further recognize the feature of differential genes, we performed GO analysis on DEGs through the DAVID website [3]. We found that DEGs are mostly enriched in the cellular component of cell fraction, soluble fraction, extracellular space, extracellular region, extracellular region part, cell projection membrane, complement, component C1 complex, brush border membrane, occluding junction and tight junction (Fig. 2B). With regard to biological process (BP), the DEGs are involved in the modulation of anion transport, reaction to drug, gluconeogenesis, reaction to incretion stimulus, aging, hexose biosynthetic process, reaction to endogenous stimulus, modulation of fibrinolysis, modulation of blood coagulation and reaction to steroid hormone stimulus (Fig. 2A). The changes in molecular function were expressively associated with anion transmembrane transporter activity, organic anion transmembrane transporter activity, oxidoreductase activity, cofactor binding, oxidoreductase activity, anion : anion antiporter activity, identical protein binding, antiporter activity, phosphoinositide phospholipase C activity and phospholipase C activity (Fig. 2C). Then we analyzed the KEGG pathway using the CLUGO plug‐in in cytoscape software [4]. We identified that DEGs are largely engaged into glycolysis; complement and coagulation cascades; valine, leucine and isoleucine degradation; prion disease; phenylalanine, tyrosine and tryptophan biosynthesis; inositol phosphate metabolism; and drug metabolism (Fig. 2D).

### PPI network analysis and screening for hub genes

**Fig. 3:**
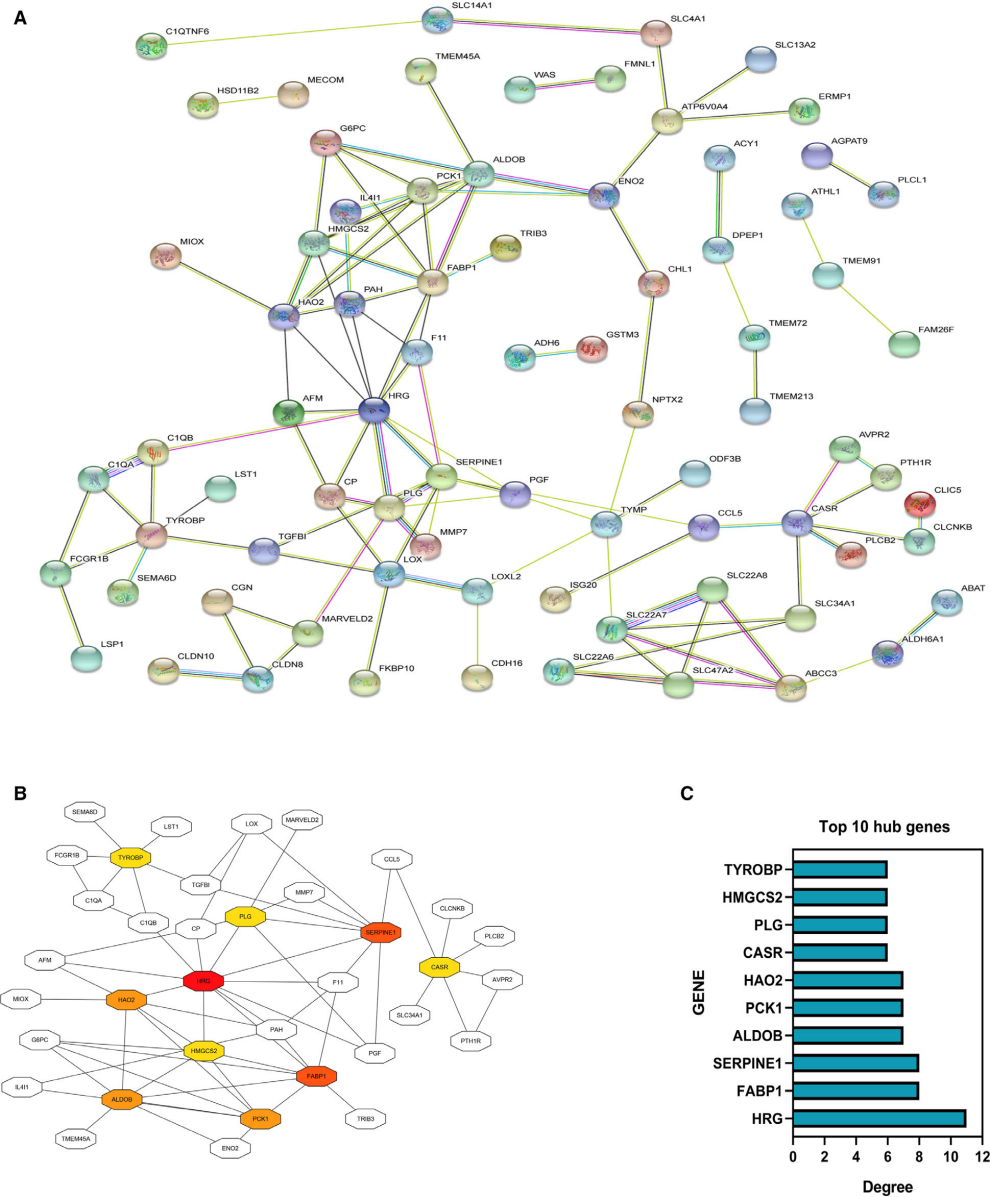
PPI network analysis and screening for hub genes. (A) PPI analysis of 122 DEGs through STRING. (B) Identifying hub genes according to the degree score by means of cytoHubba plug‐in. (C) The top 10 hub genes were finally selected.

STRING is an online tool for studying and integrating the interactions between proteins [6]. To study the relationship among various DEGs, we put 122 DEGs into STRING for PPI analysis, and we can get the connection among genes (Fig. 3A). The top 10 genes, *HRG*, *FABP1*, *SERPINE1*, *ALDOB*, *PCK1*, *HAO2*, *CASR*, *PLG*, *HMGCS2* and *TYROBP*, were confirmed as potential hub genes according to the degree score generated by cytoHubba plug‐in in cytoscape software (Fig. 3B,C)[18].

### Differential expression and survival analysis of Hub genes in ccRCC

**Fig. 4:**
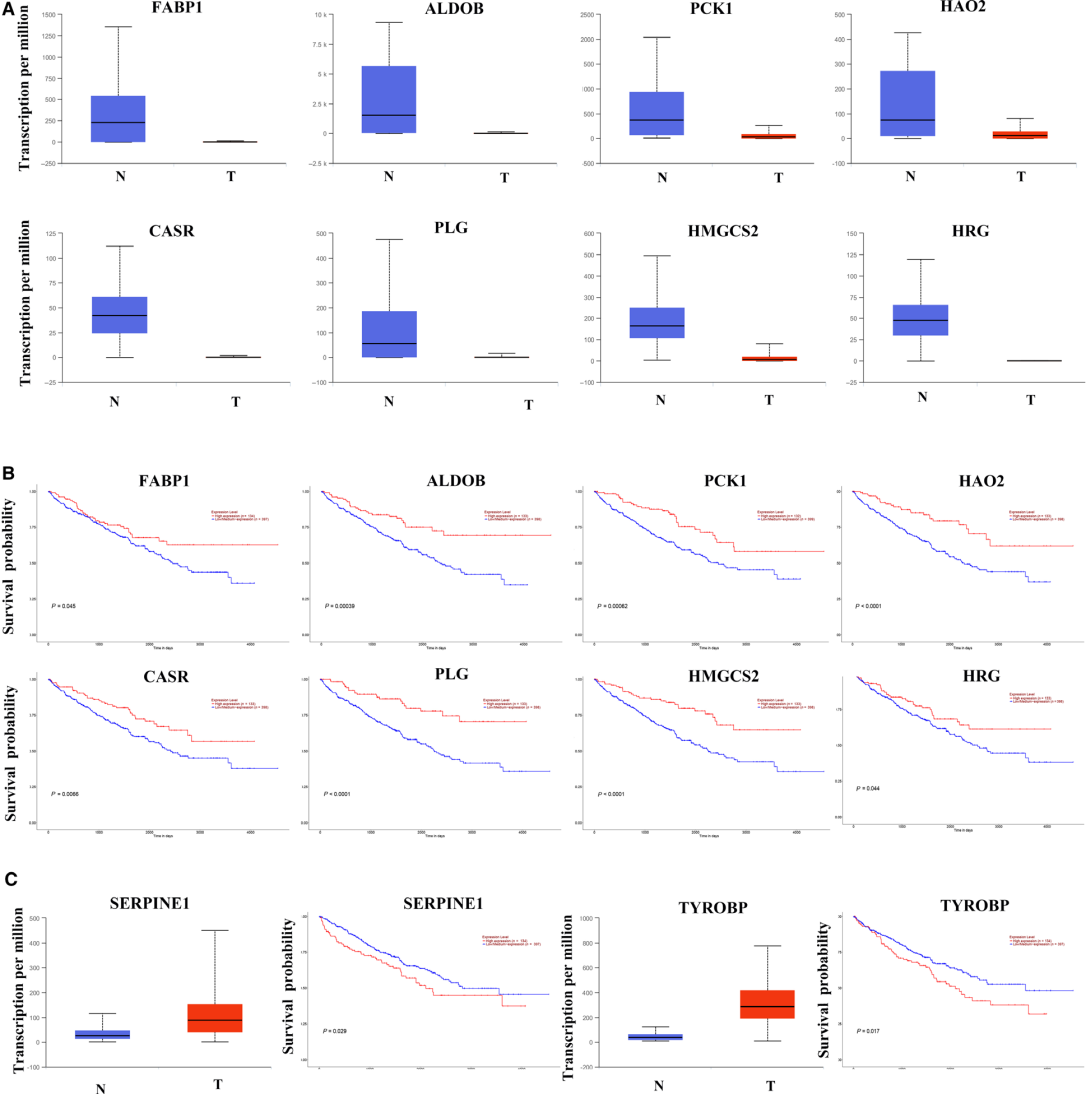
Differential expression and survival analysis of hub genes in ccRCC. (A) Analysis of the difference in expression of eight down‐regulated hub genes between ccRCC and normal kidney tissues via the UALCAN website. (B) Survival analysis of eight down‐regulated hub genes between ccRCC and normal kidney tissues via the UALCAN website. The red survival curve represents the high‐expression group, and the blue survival curve represents the low‐expression group. (C) Differential expression analysis and survival analysis of two up‐regulated hub genes between ccRCC and normal kidney tissues via the UALCAN website. The red survival curve represents the high expression group, and the blue survival curve represents the low expression group.

To confirm the differential expression of the hub gene between ccRCC and normal kidney tissues, we validated the 10 hub genes using the UALCAN website [1]. We found that *HRG*, *FABP1*, *ALDOB*, *PCK1*, *HAO2*, *CASR*, *PLG* and *HMGCS2* were down‐regulated in ccRCC in comparison with normal kidney tissues (Fig. 4A), and these eight genes were exposed to poor survival rate (Fig. 4B). Although the expression of *SERPINE1* and *TYROBP* was expressively up‐regulated, the up‐regulation of *SERPINE1* and *TYROBP* was linked to poor prognosis of ccRCC (Fig. 4C). From the earlier results, it can be inferred that *HRG*, *FABP1*, *ALDOB*, *PCK1*, *HAO2*, *CASR*, *PLG* and *HMGCS2* may be the tumor suppressor genes of ccRCC, whereas *SERPINE1* and *TYROBP* may be the oncogenes of ccRCC.

### ONCOMINE database validates hub genes

**Fig. 5:**
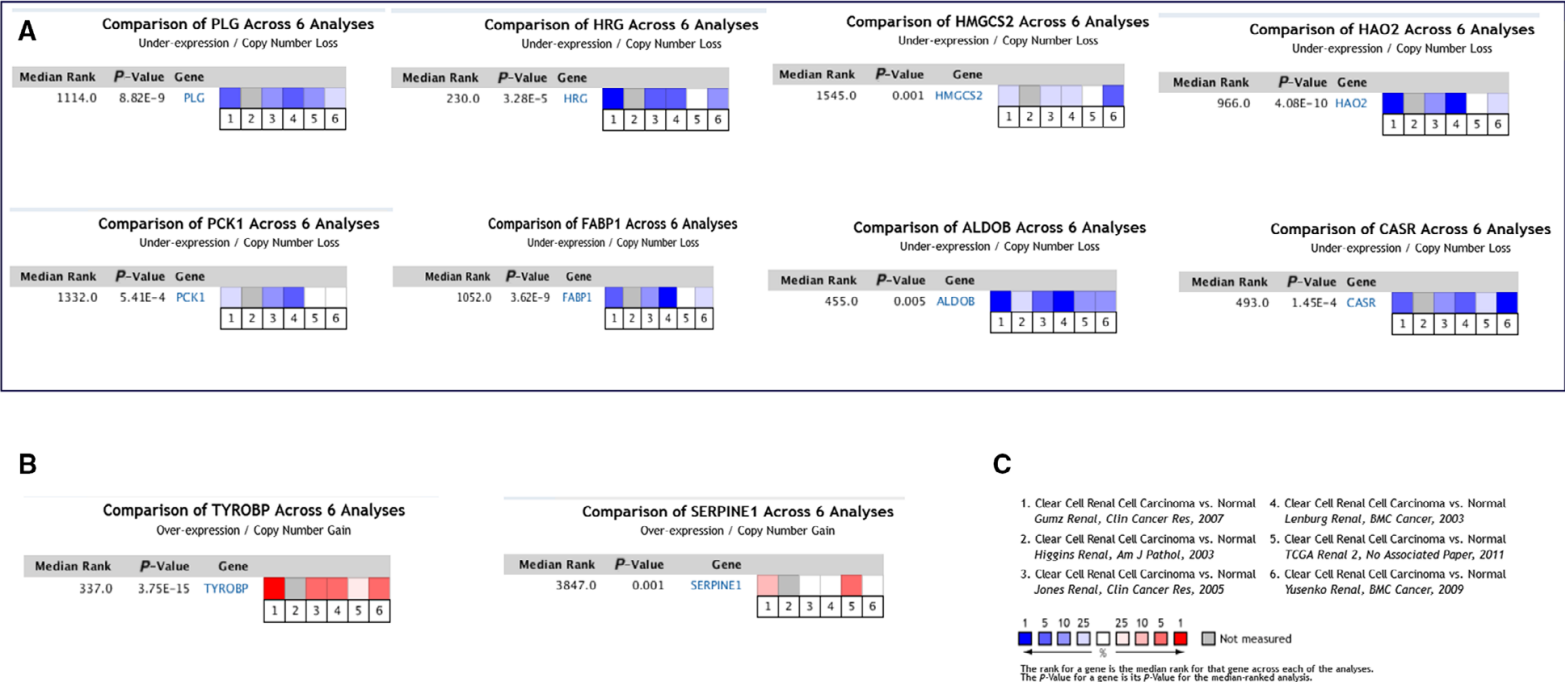
Oncoming database to reverify hub genes (we used the paired *t*‐test for statistical analysis, and *P* < 0.05 was considered statistically significant). (A) Revalidation of eight down‐regulated genes using the ONCOMINE database. (B) Revalidation of two up‐regulated genes using the ONCOMINE database. (C) Meta‐analysis of six different databases in the ONCOMINE database.

To further demonstrate the reliability of the Hub genes we screened, we used the ONCOMINE database to reverify the hub genes [2]. We selected six qualified databases from the ONCOMINE database and conducted a meta‐analysis of 1312 tissues. Consistently, *HRG*, *FABP1*, *ALDOB*, *PCK1*, *HAO2*, *CASR*, *PLG* and *HMGCS* expressions were significantly down‐regulated (Fig. 5A), whereas *SERPINE1* and *TYROBP* expressions were up‐regulated (Fig. 5B); the results of meta‐analysis revealed a statistically significant difference (Fig. 5C).

### *TYROBP* and *HRG* are the two promising candidate genes in ccRCC

**Fig. 6:**
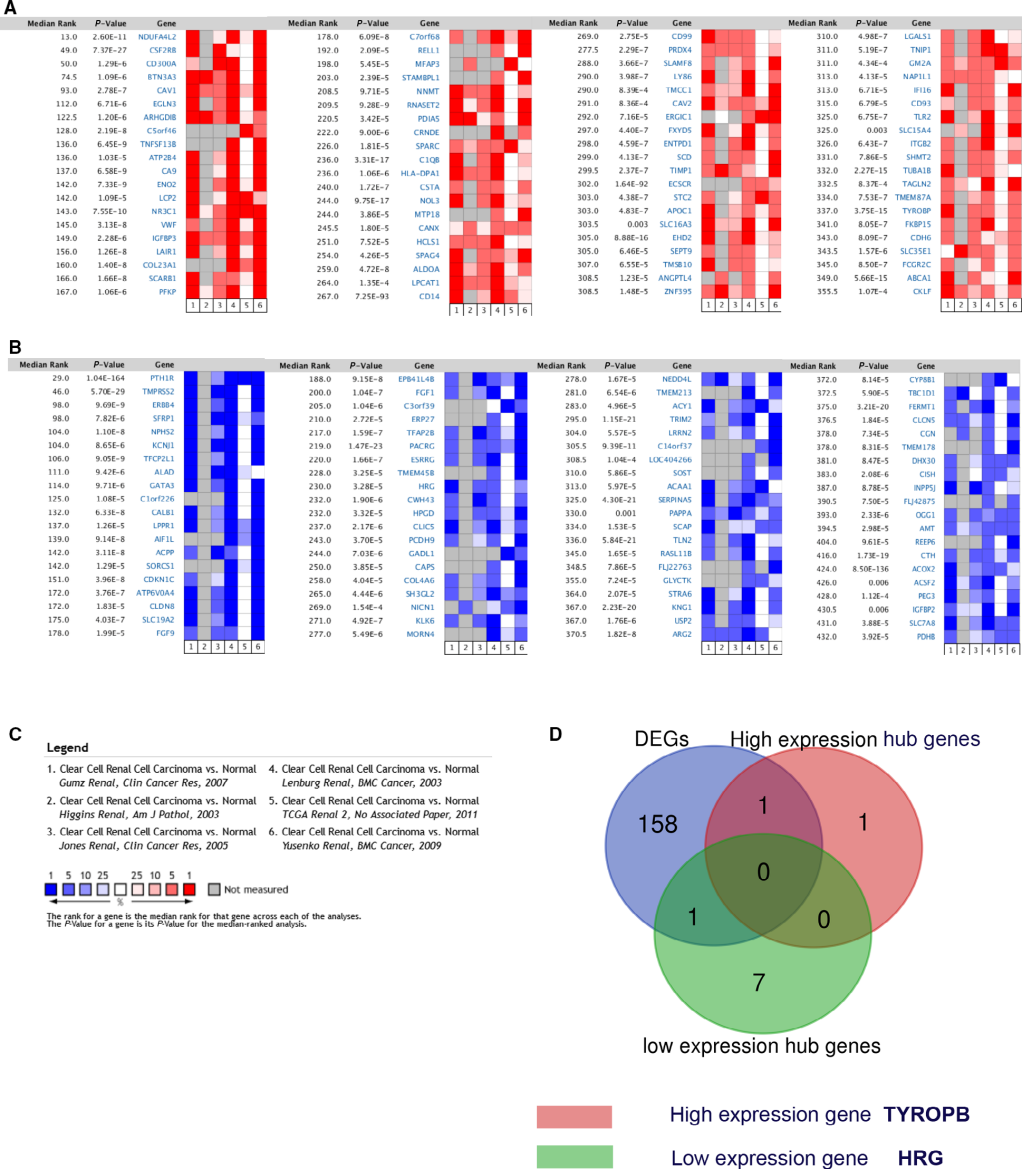
*TYROBP* and *HRG* are the promising candidate genes in ccRCC (we used the paired *t*‐test for statistical analysis, and *P* < 0.05 was considered statistically significant). (A) Top 80 up‐regulated genes were screened from six databases from the ONCOMINE database. (B) Top 80 down‐regulated genes were screened from six databases from the ONCOMINE database. (C) Meta‐analysis of six different databases in the ONCOMINE database. (D) Interpretation of selected 160 genes and 10 hub genes to identify possible candidate genes using Venn diagram; *TYROBP* with high expression and *HRG* with low expression were selected.

We selected six eligible databases from the ONCOMINE database and then performed a meta‐analysis to screen out the top 80 up‐regulated genes and the top 80 down‐regulated genes (Table 2 and Fig. 6A–C). Then, by using the Venn diagram, we analyzed the 160 screened genes and 10 hub genes. Finally, *TYROBP* and *HRG* were identified as possible candidate genes (Fig. 6D).

**Table 2:** A total of 160 DEGs were identified from ONCOMINE datasets.

DEGs	Gene names
Up‐regulated (*n* = 80)	*NDUFA4L2*, *CSF2RB*, *CD300A*, *BTN3A3*, *CAV1*, *EGLN3*, *ARHGDIB*, *C5ORF46*, *TNFSF13B*, *ATP2B4*, *CA9*, *ENO2*, *LCP2*, *NR3C1*, *VWF*, *IGFBP3*, *LAIR1*, *COL23A1*, *SCARB1*, *PFKP*, *C7ORF68*, *RELL1*, *MFAP3*, *STAMBPL1*, *NNMT*, *RNASET2*, *PDIA5*, *CRNDE*, *SPARC*, *CAQB*, *HLA‐DPA1*, *CSTA*, *NOL3*, *MTP18*, *CANX*, *HCLS1*, *SPAG4*, *ALDOA*, *LPCAT1*, *CD14*, *CD99*, *PRDX4*, *SLAMF8*, *LY86*, *TMCC1*, *CAV2*, *EGRGIC1*, *FXYD5*, *ENTPD1*, *SCD*, *TIMP1*, *ECSCR*, *STC2*, *APOC1*, *SLC16A3*, *EHD2*, *SEPT9*, *TMSB10*, *ANGPYL4*, *ZNF395*, *LGALS1*, *TNIP1*, *GM2A*, *NAPIL1*, *IFIL6*, *CD93*, *TLR2*, *SLC15A4*, *ITGB2*, *SHMT2*, *TUBA1B*, *TAGLN2*, *TMEM87A*, *TYROBPFKP15*, *CDH6*, *SLC35EA*, *FCGR2C*, *ABCA1*, *CKLF*
Down‐regulated (*n* = 80)	*PTH1R*, *TMPRSS2*, *ERBB4*, *SFRP1*, *NPHS2*, *KCNJI*, *TFCP2L1*, *ALAD*, *GATA3*, *C1ORF226*, *CALB1*, *LPPR1*, *AIF1L*, *ACPP*, *SORCS1*, *CDKN1C*, *ATP6VOA4*, *CLDN8*, *SLC19A2*, *FGF9*, *EBP41L4B*, *FGF1*, *C3ORF39*, *ERP27*, *TFAP2B*, *ACRG*, *ESRRG*, *TMEM45B*, *HRG*, *CWH43*, *HPGD*, *CLICS*, *PCDH9*, *GADL1*, *CAPS*, *COL4A6*, *SH3GL2*, *NICN1*, *KLK6*, *MORN4*, *NEDD4L*, *TEME213*, *ACY1*, *TRIM2*, *LRRN2*, *C14ORF37*, *LOC404266*, *SOST*, *ACAA1*, *SERPINAS*, *APPA*, *SCAP*, *TLN2*, *RASL11B*, *FLJ22763*, *GLYCTK*, *STRA6*, *KNG1*, *USP2*, *ARG2*, *CYP8B1*, *TBC1D1*, *FERMT1*, *CLCNS*, *CGN*, *TEMED178*, *DHX30*, *CISH*, *NPP5J*, *FLJ42875*, *OGG1*, *AMT*, *REEP6*, *CTH*, *ACOX2*, *ACSF2*, *PEG3*, *IGFBP2*, *SLC7A8*, *PDH8*

### *TYROBP* may play an important role as an oncogene in the progression of ccRCC

**Fig. 7:**
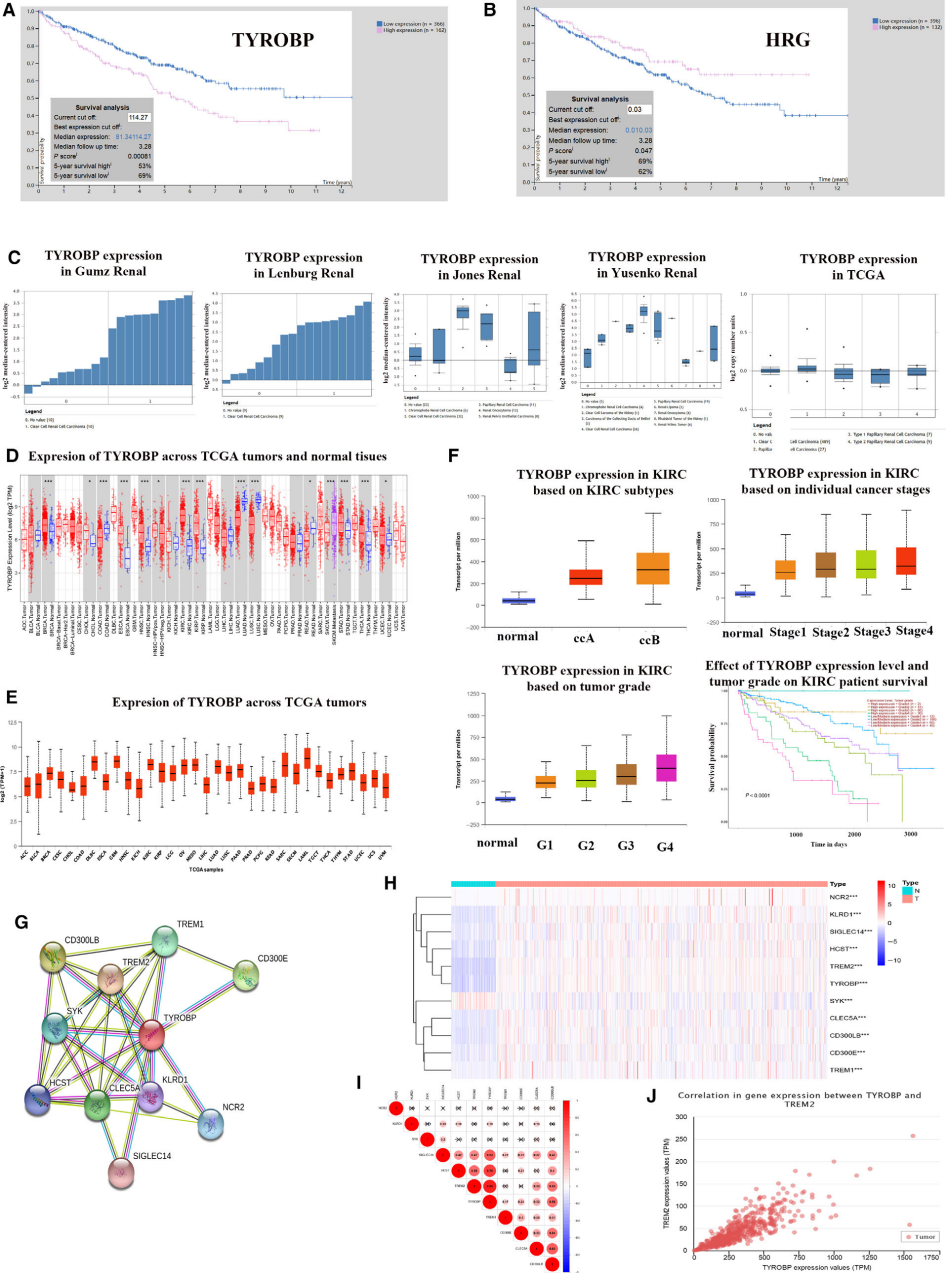
*TYROBP* was identified as a promising candidate gene (we used the paired *t*‐test for statistical analysis, and *P* < 0.05 was considered statistically significant). (A) Survival analysis of *TYROBP* in ccRCC by HUMAN Protein Atlas website. (B) Survival analysis of *HRG* in ccRCC by HUMAN Protein Atlas website. (C) *TYROBP* is up‐regulated in various databases through ONCOMINE database. (D) Analysis of the difference of expression of *TYROBP* between ccRCC and normal kidney tissues in multiple tumors through the TIMER website (*P < 0.05, ***P < 0.001). (E) Analysis of the difference in expression of *TYROBP* in various tumors. (F) The relationship between *TYROBP* expression and subtype, grade, stage and survival analysis of ccRCC. (G) Screening for genes that have a PPI network with *TYROBP* through the STRING website. (H) Analysis of the expression of *TYROBP* and the genes that interact with *TYROBP* in normal tissues and tumor tissues by r language analysis and visualization through the pheatmap package. (I) corrplot package of the r language to analyze the coexpression of *TYROBP* and genes that interact with *TYROBP*. (J) *TREM2* with high coexpressed level with *TYROBP* by corrplot package of the r language.

To find a gene that is most important in the occurrence and progression of ccRCC, we used the Human Protein Atlas website to conduct survival analysis on the two genes *TYROBP* and *HRG*. We discovered that for survival analysis, *TYROBP* has a higher statistical significance than *HRG*, with *P* values of 0.00081 and 0.047, respectively (Fig. 7A,B). So, we choose *TYROBP* for our future research. First of all, we found that *TYROBP* is obviously up‐regulated in various databases through ONCOMINE database analysis (Fig. 7C). Then we analyzed the TCGA database by means of the TIMER website and UALCAN website and found that *TYROBP* was up‐regulated in various tumors as oncogenes, including *ESCA*, *CBM*, *HNSC*, *KIRP* and *SARC* (Fig. 7D,E). Moreover, we analyzed the diverse subtype, stage, grade and survival of *TYROBP* in ccRCCs and kidney normal tissues; we detected that *TYROBP* expression is closely associated with the stage and grade of ccRCC, and the higher the stage or *TYROBP* expression, the lower the survival rate of ccRCCs. There is a correlation between stages and grades, where patients with higher stages have higher grades and vice versa. However, we made an interesting finding that in grade 4 ccRCC, the high *TYROBP* expression group has a better survival rate than the low *TYROBP* expression group (Fig. 7F). Here, we need further experiments to explain the reasons. To further understand the function of *TYROBP*, we found the 10 genes that are most closely related to the interaction of *TYROBP* protein through the STRING website (Fig. 7G) [6]. We found that *TYROBP* and genes that interact with *TYROBP* are differentially expressed in KIRC by downloading the relevant data of KIRC in the TCGA database, including 72 normal samples and 539 tumor samples, where the expressions of *NCR2*, *KLRD1*, *SIGLEC14*, *HCST*, *TREM2*, *TYROBP*, *TREM1*, *CD300E*, *CLEC5A* and *CD300LB* are higher in tumors than in normal kidney tissue, and the expression of *SYK* is lower in tumors than in normal kidney tissue (Fig. 7H). In further coexpression analysis, we found that *TYROBP* and the genes that have a protein interaction relationship with *TYROBP* have a clear coexpression relationship, and the gene most closely related to *TYROBP* is *TREM2* (Fig. 7I,J).

### TYROBP is up‐regulated in ccRCC samples

**Fig. 8:**
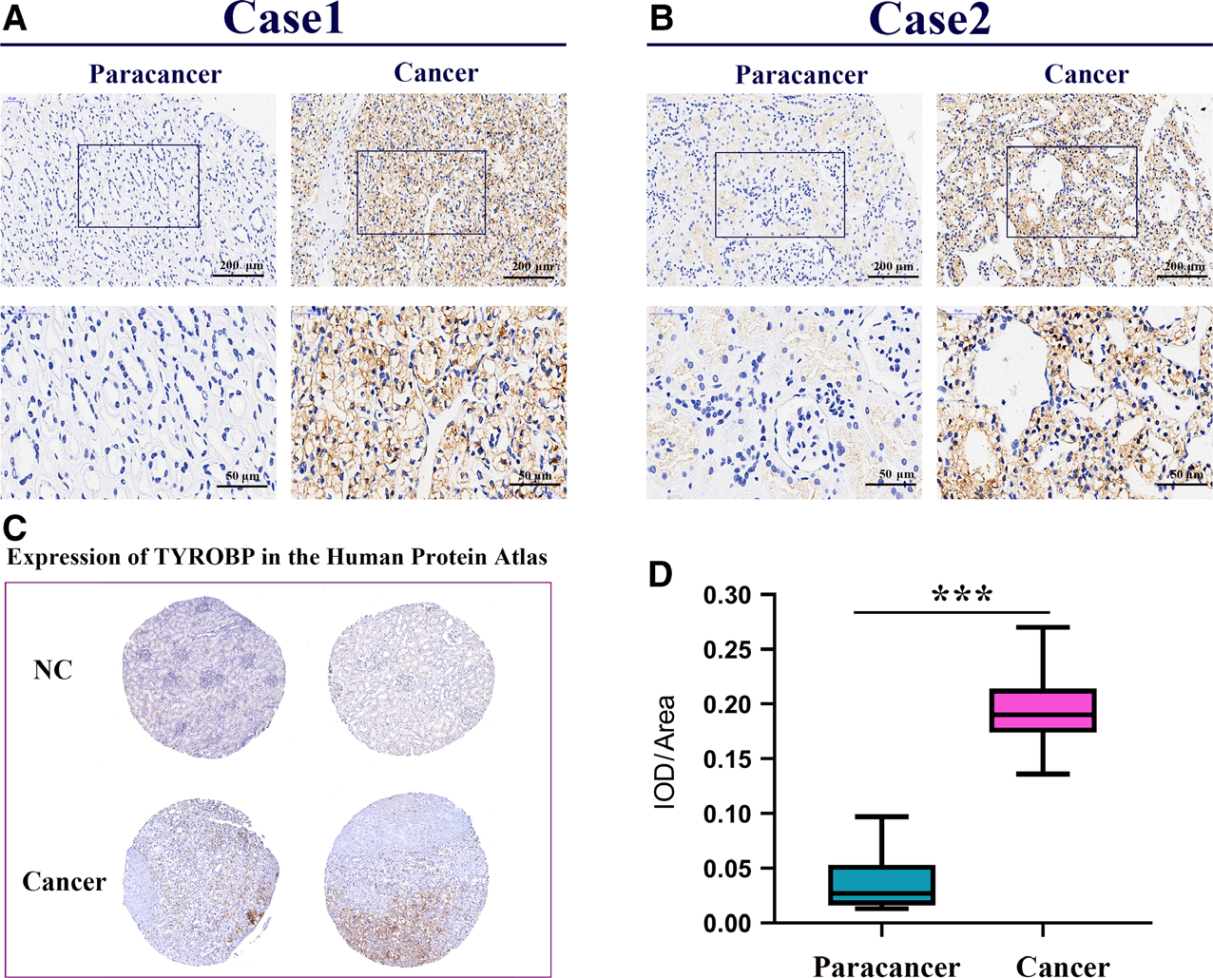
TYROBP is up‐regulated in ccRCC samples compared with renal tissues. We used the paired *t*‐test for statistical analysis, and *P* < 0.05 was considered statistically significant (we selected 10 samples for testing and repeated three times). (A) IHC analysis of TYROBP in ccRCC tissue and paracarcinoma (case 1); scale bars: 200 and 50 μm, respectively. (B) IHC analysis of TYROBP in ccRCC tissue and paracarcinoma (case 2); scale bars: 200 and 50 μm, respectively. (C) Immunohistochemical images of TYROBP in kidney cancer and normal tissues obtained from Human Protein Atlas. (D) Integrated optical density/area analysis was performed using imagepro plussoftware, and the statistics were performed by graphpad prism 8 software ( ***P < 0.001).

To further evaluate the expression of TYROBP, we performed IHC analysis to assess the protein levels of TYROBP in 15 ccRCCs and matched paracarcinoma samples (Fig. 8A,B). The expression of TYROBP was expressively up‐regulated in ccRCC in comparison with the adjacent normal tissues (Fig. 8D). In addition, the protein expression level of TYROBP was evaluated based on the Human Protein Atlas. Compared with normal tissues, the expression of TYROBP in ccRCC tissues was significantly higher, which is consistent with our research (Fig. 8C).

**Fig. 9:**
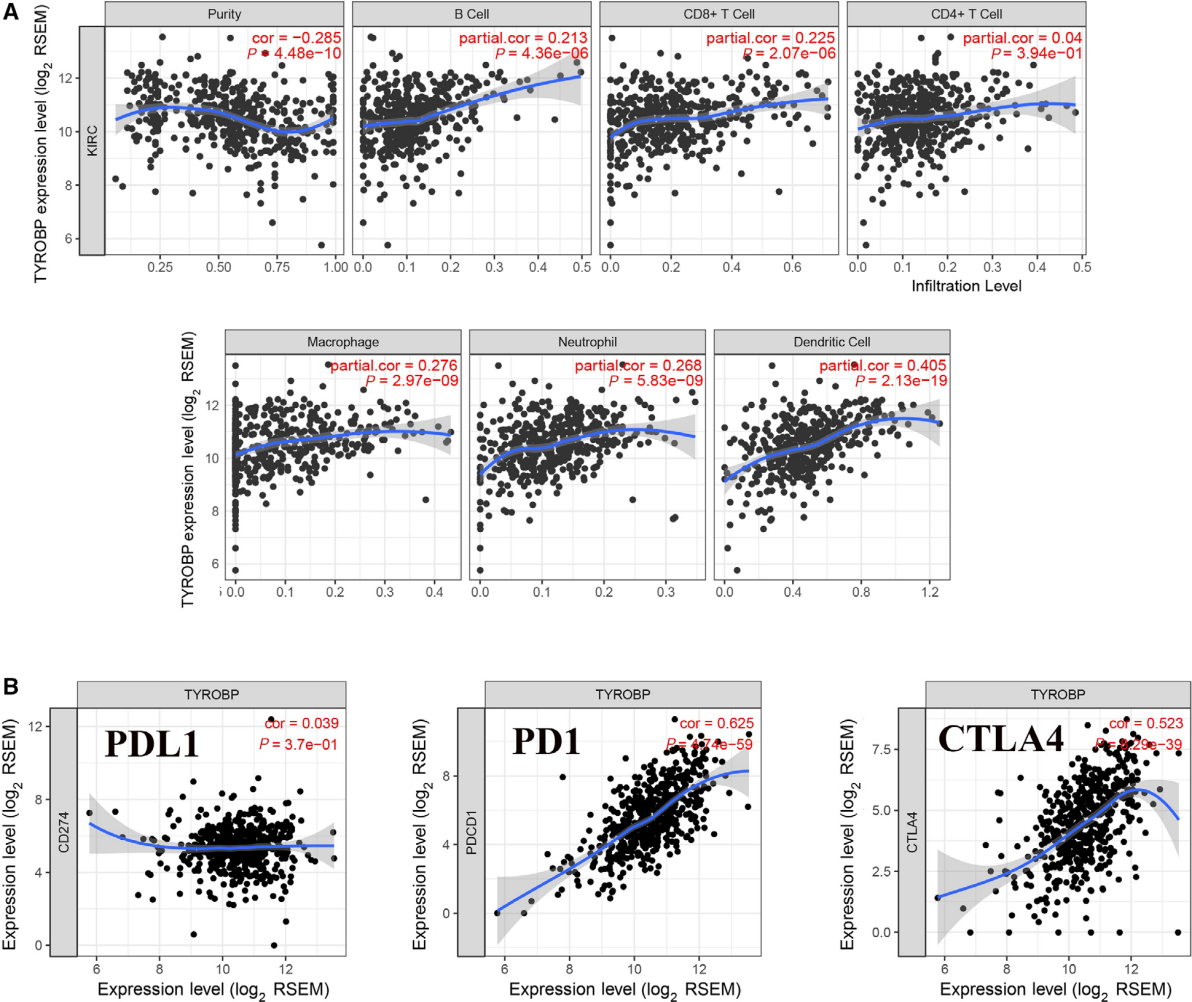
*TYROBP* was identified as having a connection with immune cells and immune checkpoint‐related gene. (A) The relationship between *TYROBP* and immune cells infiltration was analyzed by the TIMER website. (B) The relevance between *TYROBP* and *PD‐1*, *PDL‐1* and *CTLA‐4* was analyzed by immunological correlation analysis.

*TYROBP* is identified as a connection with immune cells infiltration and immunological checkpoint‐related gene. We analyzed the connection between *TYROBP* and immune cells infiltration, and the results showed the high expression of *TYROBP* is linked to higher infiltration rate in immune cells, containing B cells, CD8^+^ T cells, CD4^+^T cells, macrophage, neutrophil and dendritic cells (Fig. 9A). Furthermore, we observed the relevance between *TYROBP* and *PD‐1*, *PDL‐1* and *CTLA‐4* expression, and we found the *TYROBP* is coexpressed with *PD‐1* and *CTLA‐4*, but has no connection with *PDL‐1* (Fig. 9B).

## Discussion

ccRCC is biologically heterogeneous and has variable clinical processes, and no predictive biomarkers have been validated in ccRCC [18, 19]. However, because the bioinformation analysis developed rapidly in recent decades, we can quickly and accurately find new biological and therapeutic biomarkers. Results from bioinformatics analysis studies have found some potential targets for the treatment of ccRCC. However, the small number of samples limits the accuracy [20, 21]. In our study, we combined a large sample size and multiple analytical methods to explore novel therapeutic targets. We identified the top 250 significant DEGs by TCGA database and performed survival analysis for each gene. Among them, 55 up‐regulated and down‐regulated DEGs were screened for poor prognosis. GO analysis is widely used as a gene enrichment assay. These significant DEGs‐related GO analyses indicated that they are closely related to cancer biological behaviors, such as gluconeogenesis modulation of fibrinolysis and coagulation. KEGG revealed that DEGs are largely engaged into glycolytic/gluconeogenesis, peroxisome proliferator‐activated receptor alpha (PPAR) signaling pathway, and the complement and coagulation cascades. This indicated that ccRCC is closely related to metabolism and immunity regulation [22, 23].

Many research studies have shown that carcinogenesis may be strongly linked to metabolism, which confirmed the general direction of our research as well [24, 25]. Increased glycolysis is a hallmark of malignancy and is associated with invasiveness and poor prognosis. Tumor cells use aerobic glycolysis to meet energy and membrane structure requirements to achieve the Warburg effect, especially in ccRCCs [26]. For further systemic analysis of the relationship and functions of important DEGs in ccRCC, we obtained 10 DEGs with the highest degree for further study. The survival analysis showed that eight down‐regulated hub genes (*HRG*, *FABP1*, *ALDOB*, *PCK1*, *HAO2*, *CASR*, *PLG*, and *HMGCS2*) and two up‐regulated hub genes (*SERPINE1* and *TYROBP*) may be the key genes in ccRCC. Interestingly, most of these hub genes are metabolic or immune‐related genes, and plenty of them have been well investigated in ccRCC or other tumors. For example, ALDOB is an important glycolytic enzyme [27]. The down‐regulation of ALDOB has been reported in ccRCC tissue compared with kidney tissue, which is consistent with our results. The loss of ALDOB was significantly correlated with a worse Heng prognostic score and a lower 2‐year survival rate in renal cell carcinoma [28]. *HAO2* inhibits the malignancy of ccRCC by promoting the lipid catabolism process and eliminating lipid accumulation [29]. SERPINE1, also known as Plasminogen activator inhibitor‐1(PAI‐1), is observed to be overexpressed in many cancer types associated with poor prognosis. In ccRCC, high expression of PAI‐1 is closely related to aggressive characteristics, including high nuclear grade and distant metastasis [30]. *FABP1* is involved in the modulation of various cellular processes, including involvement in the modulation of inflammatory states and lipid metabolism through interacting with PPAR [31]. *FABP1* also inhibits epithelial–mesenchymal transition in different ways, thereby inhibiting cancer infiltration and metastasis in ccRCCs. *HMGCS2* is an important regulatory point in the pathway of converting acetyl‐CoA to ketone bodies. The gene was down‐regulated in 80–90% of intestinal tumors [32]. The literature indicates that *HMGCS2* acts as a transcription factor that coupled with PPAR, leading to *Src* expression and activation in a metabolically independent manner. These reports are consistent with our biosignal analysis results [33].

For the sake of finding a gene that is most important in the occurrence and progression of ccRCC, we performed meta‐analysis among multiple databases from the ONCOMINE database. We obtained the top 80 high/low expressed genes and then interpreted these genes with 10 hub genes. Finally, *TYROBP* and *HRG* were identified as candidate genes. Studies have found that *HRG* inhibits tumor growth and metastasis by enforcing host–antitumor immune responses and promoting tumor vascular normalization. Mantovani and Sica [34] reported that *HRG* increased tumor infiltration by antigen‐presenting dendritic cells, cytotoxic T lymphocytes and natural killer (NK) cells to inhibit tumor growth. In addition, a previous study has identified *TYROBP* as a hub gene in ccRCC progression through weighted gene coexpression network analysis, indicating that *TYROBP* is strongly linked to clinical trait and vital BPs [35].

To better confirm the biological function of *TYROBP*, we screened a crucial oncogene, *TREM2*. A noteworthy finding was that *TREM2* not only has a significant coexpression relationship with *TYROBP* but also existed in the PPI network of TYROBP‐related genes. *TREM2* plays an important role in regulating adaptive and innate immunity by pairing with *TYROBP* [36]. According to a report, the up‐regulation of *TREM2* is positively related to poor prognosis in patients with gastric cancer [37].

To further confirm the relationship between candidate genes and patient survival, we performed a survival analysis, and we found that *TYROBP* was more statistically significant than *HRG*. The up‐regulation of *TYROBP* was positively related to tumor grade and patients' prognosis. In addition, significant up‐regulation of *TYROBP* was revealed in ccRCC tissues compared with normal tissues. Our research revealed that *TYROBP* plays a vital part in the occurrence and progression of ccRCC and is closely related to the prognosis of patients with ccRCC. However, we found that for grade 4 ccRCC, the high *TYROBP* expression group had a better survival rate than the low *TYROBP* expression group. This phenomenon may be caused by the following reasons. First, after ccRCC was divided into eight groups, the sample size of the subgroups may be reduced, which affects the accuracy of the results. Second, according to the results of the previous bioinformatics analysis, high‐expression *TYROBP* should correspond to high‐grade samples. For high‐grade samples with low *TYROBP* expression, there may be case specificity, and analysis on the strength of this specificity may lead to bias in results. We will conduct further analysis in future clinical trials for this argument. KEGG results indicated that the genes related to the target gene *TYROBP* were enriched in NK cell‐mediated cytotoxicity antigen processing, presentation and B‐cell receptor signaling pathway. TYROBP, also known as KARAP/DAP12 (killer cell activating receptor‐associated protein/DNAX activating protein of 12 kDa), is an ITAM (immunoreceptor tyrosine‐based activation motif) that mainly expressed in natural killer cells and myeloid cells, where it can bind to several immunoreceptors contributing to a variety of biological functions [38]. In the experiment with *TYROBP* knockout mice, it was pointed out that NKG2D, a DAP12‐dependent NK cell receptor, is involved in antitumoral activity and regulates NK cell function, respectively [39, 40]. NKP44 is another DAP‐12‐dependent receptor expressed on the surface of human NK cells and plays an important part in the recognition and elimination of tumor cells [41]. These are all consistent with our findings that *TYROBP* and related genes are mainly engaged in immunoregulatory mechanisms, such as NK cell‐mediated cytotoxicity. To further identify the mechanism of *TYROBP* expression in the development of ccRCC, we analyzed the connection of *TYROBP* expression levels with immune cells infiltration, and we discovered that *TYROBP* is related to high infiltration rate in immune cells, including B cells, CD8^+^ T cells, CD4^+^ T cells, macrophages, neutrophils and dendritic cells. Research has shown that *TYROBP* could play a vital part in the immune cell infiltration through a variety of ways. For instance, the expression of DAP12 in tissue‐resident alveolar macrophages mediates acute noninfectious tissue damage by regulating neutrophil trafficking [42]. In hypoxic mature dendritic cells (mDCs), *TREM1* gene promotes the secretion of proinflammatory cytokines and chemokines by activating DAP12‐related signal pathways [43]. Siglec‐15 promotes the secretion of transforming growth factor‐β in tumor‐associated macrophages through the DAP12–Syk pathway, which promotes tumor progression through regulating the tumor microenvironment [44]. We know that the progression of cancer and its response to treatment are influenced by both innate and adaptive immunity, and these immune‐related cells play an important regulatory role in the occurrence and development of tumors. *TYROBP* probably promotes tumor progression by interacting with immune cells. It has recently been reported that cell fusion contributes to cancer spreading, and *TYROBP* is essential for macrophage fusion. Tumor cells in the bone microenvironment stimulate the recruitment and activation of osteoclasts and osteoblasts. The tumor microenvironment not just promotes the tumor proliferation but also determines the perfused site of metastasis. It has been found that *TYROBP* is up‐expressed in breast cancer cells and is significantly related to skeletal metastasis and poor prognosis [45]. *TYROBP*/*ITAM* pathway may be involved in bone metastasis of breast cancer. According to reports, the possibility of bone metastasis of ccRCC in the form of osteoclasts is very high. Whether the high expression of *TYROBP* is related to the bone metastasis of ccRCC is the direction of our further research.

‘Cancer immunotherapy’ was named as 2013’s breakthrough of the year by science [46]. *CTLA‐4*, *PD‐1* and *PDL‐1* inhibitors are among the most effective immunotherapy methods for cancer treatment [47]. ccRCC has also been shown as an immune tumor with a synergistic effect of angiogenesis and immunosuppression, and its growth is closely related to tumor immunity. Many studies have shown that *PD‐1* and *PDL‐1* were predominantly expressed in the tumor microenvironment of high‐grade ccRCC tissues. *CTLA‐4* is expressed only in T cells; *PD‐1* is expressed in activated T cells, B cells and NK cells; and high expressed *PD‐1* or *CTLA‐4* are linked to invasiveness and poor prognosis of ccRCC [48]. Our target gene has a correlation with *PD‐1*, *CTLA‐4* and overexpression of *TYROBP* related to the high stage and worse outcome of the patient with ccRCC. Currently, a *PD‐1* and *PDL‐1* antibody has been introduced for the treatment of advanced ccRCC and has achieved an effective result [49]. *TYROBP* is likely to be the newly discovered oncogene involved in the immune regulation mechanism and is promising as an important gene target for ccRCC diagnosis and immunotherapy.

## Conclusions

We processed a series of bioinformatics analyses to seek ccRCC‐related hub genes; after repeated multiple validations, *TYROBP* was identified as the potential prognostic biomarker. The high expression of *TYROBP* is obviously related to the low survival rate. This gene is also involved in the immune regulation system and has a coexpression relationship with *PD‐1* and *CTLA‐4*. This give us a new idea for further investigation.

## Conflict of interest

The authors declare no conflict of interest.

## Author contributions

GW designed the study. TX, JW and RL carried out data acquisition and analysis. PW wrote the manuscript. GW contributed to preparing and making figures. All authors read and approved the final manuscript.

## Data Availability

The article data will be available from the corresponding author on reasonable request.
